# Eye Movements during Reading and their Relationship to Reading Assessment Outcomes in Swedish Elementary School Children

**DOI:** 10.16910/jemr.15.4.3

**Published:** 2022-10-13

**Authors:** Andrea Strandberg, Mattias Nilsson, Per Östberg, Gustaf Öqvist Seimyr

**Affiliations:** The Marianne Bernadotte Centre, Department of Clinical Neuroscience, Karolinska Institutet, Sweden; Department of Clinical Science, Intervention and Technique, Karolinska Institutet, Sweden

**Keywords:** Eye movement, eye tracking, reading, assessment, individual differences, reading development

## Abstract

The characteristics of children’s eye movements during reading change as they gradually become better readers. However, few eye tracking studies have investigated children’s reading and reading development and little is known about the relationship between reading- related eye movement measures and reading assessment outcomes. We recorded and analyzed three basic eye movement measures in an ecologically valid eye-tracking set-up. The participants were Swedish children (n = 2876) who were recorded in their normal school environment. The relationship between eye movements and reading assessment outcomes was analyzed in using linear mixed effects models. We found similar age-related changes in eye movement characteristics as established in previous studies, and that eye movements seem to correlate with reading outcome measures. Additionally, our results show that eye movements predict the results on several tests from a word reading assessment. Hence eye tracking may potentially be a useful tool in assessing reading development.

## Introduction

Eye tracking has been a valuable tool in many fields of research during the last decades, including medicine, psychology and education (13; 14; 26; 37; 45; 55). It is frequently used to study reading and reading development. Eye tracking has enabled researchers to investigate the underlying mechanisms of text processing (4; 17; 24; 28; 32; 50), as it provides a real-time measure and captures small variations in how the linguistic stimuli is processed in the brain (see review by 8; 7; 29; 33; 54). While a significant amount of eye movement research has described the reading process in skilled adult readers, less has been dedicated to reading and its development in children. However, as pointed out by many researchers (35) there are coherent, developmental differences between the characteristics of eye movements in beginning and skilled readers. As an individual’s reading skill develops, changes occur in the interaction between cognitive, visual and oculomotor processes (35; 4). These changes are consistent across the education systems, orthographies and languages that have been examined to this date (35). The term fixation describes the act of fixating the fovea on a given spot, where the eye remains relatively still for at least 60-80 ms when reading. It is the minimal amount of time that an adult reader needs to visually encode a printed word, for further processing to proceed (3). However, an average fixation is approximately 250 ms in adult readers. Fixations during reading are usually analysed in terms of their temporal duration and frequency of occurrence. Saccades refer to the rapid movements of the eye that occur in-between fixations. Saccades during reading are commonly analysed in terms of their spatial length and frequency of occurrence. Eye movements in the reading direction (from left to right in alphabetical orthographies) are called progressive or forward saccades while saccades from right to left are referred to as regressive saccades or regressions (32). In adults, forward saccade amplitude is around 4-5 degrees (40) and the probability of a saccade being a regression is approximately 15-20% during normal text reading. Aspects of the recording technique, the experimental approach and the eye movement measures of interest vary depending on the scope of the study and are often partly determined by the sample age span. In the eye movement research field, researchers often use a restrained study design, in order to limit the scope of the investigation and study a certain phenomenon with high precision. In some cases, findings may be hard to translate to the world outside of the lab and thus diminish the ecological validity of the results.

A study using a moving-window paradigm by Rayner (31) exemplifies the general developmental changes in eye movement characteristics from child- to adulthood. Children of 7-8, 9-10 and 11-12 years and adults were compared and results demonstrated an increase in reading speed and saccade length with increasing age, while the mean fixation duration and number of regressions decrease. The results are consistent with previous findings (46), and have been replicated in several studies (3; 5; 6, 25; 32; 40; 44).

Some hypotheses propose that oculomotor development and maturity act as driving mechanisms behind differences in eye movement behaviour in children. According to the visual/oculomotor view, eye movements are to some extent independent from linguistic processing. The prevailing linguistic/cognitive theory attributes eye movement behaviour during reading to underlying lexical processing (32). In line with this position, changes in eye movements during the first years of reading acquisition reflect improved reading skill rather than oculomotor maturation (6; 3; 4; 35; 30, 31). However, these perspectives do not have to be mutually exclusive, but each emphasize different contributions to the reading process (57).

Individuals with reading difficulties (RD) have longer fixation durations, a higher number of fixations (and lower percentage non-fixated words) and shorter saccades compared to subjects with typical reading development. Moreover, they make a larger number of unexpected vertical gaze movements when reading multiline texts as well as fewer forward saccades than controls (15). All of these characteristics are indicative of decoding difficulties (42). The eye movements of children with RD rather resemble those of reading-level, i.e. younger, than age-matched controls. Similarly, slow readers have smaller perceptual spans compared to fast readers (9; Eden et al., 1994; 32; 34; 36). Using machine learning and recordings of reading eye movements, Nilsson et al. (27) were able to identify children in third grade with increased risk of reading difficulties with high accuracy (95.3% ± 4.6%). Thus, eye movement measures of reading are interesting not only because they allow a deeper understanding of underlying text processing, but they may be of clinical predictive value as well.

Assessment of reading ability is usually performed using various tests of component skills. Phonemic awareness, a component of phonological awareness, is of special importance for early decoding ability (see Shuele & Boudreau, 39, for a review). Phonemic awareness can be described as comprehending that language consists of various, segmented sounds that can be manipulated and combined and that the sounds are represented by letters in written language. It’s based on the alphabetic principle and has a causal connection to reading ability, supported by intervention studies reporting improved spelling, word identification and general reading ability after specific instructions and practice of phonological awareness and letter-sound knowledge (2; 10; 39; 51; 48). Phonemic awareness is assessed in various manners. In reading assessment, pseudo word decoding tasks can provide information on the strength of the connection between the phonological system and the printed representations (letters), without access to orthographic clues. According to Kamhi & Catts (22), a satisfactory assessment of a child’s word recognition ability should, beyond phonemic decoding (e.g., pseudo word reading), always include tests of word reading accuracy and reading fluency. Fluent reading can be evaluated using measures of accuracy and rate (16). The link between reading fluency, reading speed and rapid automatized naming (RAN) (see Wolf & Bowers, 56, for a review) has been evaluated continuously since the test procedure of the same name was developed by Denckla and Rudel. They found that individuals with RD have distinctively slower naming speed in a test that requires repetitive naming of objects, colours, letters and numbers in an automatic manner (Denckla & Rudel, 1976). One of the most influential accounts of what component processes that influences RAN performance claims that phonological retrieval plays a key role (52). Others accounts add visual processing and global processing speed as major contributors (56).

Certain abilities are crucial for reading and its development in children. How these abilities relate to reading eye movements is less known (4).

With few exceptions, such as Spichtig et al.'s 2016 study on reading comprehension and eye movements in North American children (n=2203), most studies to date have relatively small sample sizes due to challenges associated to recruiting under aged persons and conducting scientific experiments with very young participants. However, technical advances during the last decades have improved the possibilities within eye movement research in children.

The objective of the present study is to improve the understanding of the relationship between developmental changes in reading eye movements and reading skill. More specifically, the aim is to investigate whether the global developmental trends of children’s reading eye movements are present in data from an unrestrained and naturalistic eye tracking set-up in a large and inclusive sample. To this end, we analyse three basic eye movement variables (mean fixation duration, forward saccade amplitude and average proportion of regressions) during standard text passage reading in a sample of Swedish elementary school children. We examine how these eye movement variables relate to five different test scores, which commonly occur in Swedish word reading assessments, in three different school grades. To our best knowledge it is the first large scale study to combine eye tracking and a full word reading assessment in young children.

## Methods

The data was collected during a research project running between 2015 and 2016. Its’ purpose was to develop a screening tool for reading difficulties in children based on eye movements and machine learning. The present study makes use of the recordings originating from this project.

### Participants

The participants of this study were elementary school students in Järfälla and Trosa municipalities in Sweden. In 2020, the median yearly income was nearly the same in Trosa and Järfälla and somewhat higher than the Swedish median. The unemployment rate was higher in Järfälla than Trosa but not higher than the Swedish median percentage (38). All municipally governed elementary schools were enrolled in the study. Children who attended first and second grade in 2015 recurred next year, thus some students have been recorded twice (n = 483), which should be taken into account when considering the sample as a whole (N = 2679). However, comparisons across grades avoid allocating recordings from the same individual to the same subsample. Data was recorded during the spring semester (January-June) both years.

The authors consulted with the principals of the schools and attended parent-teacher meetings to inform caregivers and teachers about the study. Written information was distributed to the caregivers. All children in first, second and third grade were offered to participate in the study, given that written parental consent was obtained. No formal exclusion criteria were applied, as the study is population-based. No IQ testing was performed and children with Swedish as second language were included. While it is established that intellectual disability affects the ability to acquire new skills, the role of IQ in reading acquisition remains debated. Since reading skill level varies over a large span of intellectual ability, the so called discrepancy criteria between reading skill and intelligence has been questioned in contemporary research (21). Swedish children with intellectual impairment (IQ < 70) are entitled to an adjusted curriculum and syllabus within special needs schools; none were included in this sample (see table 1 for information about the sample). Both readers with Swedish as first and second language are included in the sample and cannot be identified or differentiated in the current study. However, public records testify that the proportion of students with Swedish as second language was smaller than the national average in the Trosa schools and marginally higher than the average in Järfälla (see table 2). Further, the results from the national test results give an indication of the overall reading performance in the third graders of the sample. The national tests are nationwide obligatory exams which children take for the first time in third grade, after which they reoccur in sixth and ninth grade. The purpose is to support teachers’ assessments of their students in certain subjects, in accordance to the established goals and requirements stated in the curricula.

**Figure:**
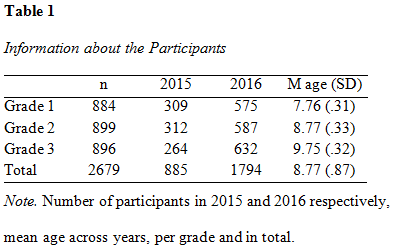


**Figure:**
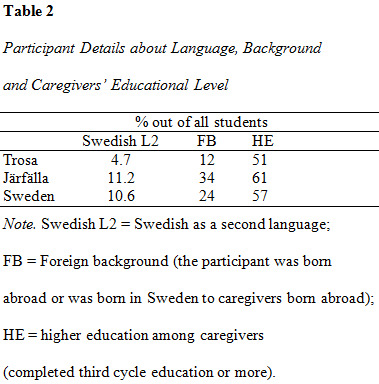


Table 3 indicates results from the reading subtests in the municipalities involved in this study, as well as the national average, in order to provide an overview of the relative educational attainment within the sample (downloadable information in English about the national test is available on the Swedish National Agency of Education’s website.

**Figure:**
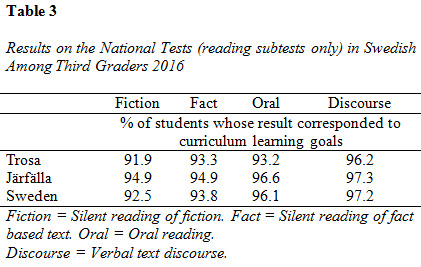


### Apparatus and Procedure

With the objective of creating a naturalistic reading situation, we employed the following strategy: 1) the stimuli for eye movement recording were normal text passages; 2) data was collected in an, to the child, familiar environment (the respective schools) and (3) an unrestrained eye tracking set-up was used. The participants completed six tasks: Letter and Word Chains (12, 20), alphabetical RAN, words and pseudo word reading and text passage reading. The procedure started with Letter and Word Chains, which participants completed individually in the classroom in accordance with the instruction handbook. No eye movement recording took place during this assignment. The remaining four tasks were presented on the eye tracking screen and completed individually together with an experiment leader in separate rooms to minimize distracting elements. Where possible, the data collected prior to termination was saved for analysis. The experiment was presented on a Tobii T120 (120 Hz; Tobii Technology AB, Danderyd, Sweden) eye tracker and run with the software Optoscope (version 3.0.0.19). The participants sat in front of the eye tracking screen at a distance of approximately 60 cm. A five-point calibration procedure was performed. Eye movements were recorded during each part of the assessment (RAN, word and pseudo word reading and text reading) but the eye movement variables used in the statistical analysis are based solely on the text reading task (averages of both texts). Sound was recorded with an external USB-microphone (Samson GoMic).

### Material

Below, the stimulus material is described in chronological order of the test procedure. See appendices for the full assignment, with the exception of Läskedjor-2 which cannot be reproduced due to copyright conditions.

#### Letter and Word Chains

Letter Chains tests children’s visual perception and motor skills by asking them to recognize and discriminate letters. It consists of 96 ten-letter-strings of mixed vowels and consonants. Each string contains two repeated letters. The child is asked to draw a line where identical letters are repeated within the same string. Word Chains tests children’s visual word recognition. It requires the participant to separate words from each other when presented in series of strings (i.e. written consecutively, without blank spaces). The test comprises 80 three-word-strings consisting of nouns, adjectives and verbs intermingled and the task is to mark the limits between words. The participants were instructed to work through as many strings as possible within the time limit of 2 minutes per sheet. Both assignments were executed in the class room setting and are part of a special edition of Läskedjor-2 (20). More information about Läskedjor is provided on the publisher’s website.

#### Rapid automatized naming

The alphabetical, serial format rapid naming task was based on the Comprehensive Test of Phonological Processing (CTOPP-II) (53), which includes subtests with naming of objects, colours, numbers and letters. Due to time restrictions, only a letter naming task was included in the present study. The characters s, a, n, c, k and t (lower-case) were presented in four rows consisting of nine items each (total of 36 items). The full set of letters is provided in appendices.

#### Word and pseudo word reading

The word and pseudo word reading task was developed by Gustaf Öqvist Seimyr in 2015 and is partly based on Test of Word Reading Efficiency (TOWRE; 49). 64 words ranging from two to nine letters were presented in rows of 8 x 8, ordered by increasing length and decreasing frequency. The pseudo words were presented in the same manner, ordered by increasing length and complexity. The pseudo words were constructed from the real words by replacing the phonemes with similar sounds according to manner and place of articulation rendered by a transposing system developed by the authors of this article. The voiced consonants n, l and m were transposed to l, m and n, respectively. Back vowels were replaced by other back vowels (a->o, o->å, å->u, u->a), and front vowels were replaced by other front vowels (e->i, i->y, y->ö, ö ->ä, ä->e), with the purpose of creating pseudo words comparable to the real words in terms of phonetic complexity. The procedure was repeated if a word was still semantically coherent after the first transposition.

The participants were instructed to read aloud as many words/pseudo words as possible within the total reading time limit of 30 seconds per sheet.

#### Text reading

The participants read two short fictional texts in Swedish and were informed that a question about the content would follow, to encourage attentive reading. They read silently when possible, but oral reading was allowed when requested by the child. In order to correspond to the varying age and reading ability of the participants, six different texts were prepared (a set of two for each grade). The texts consisted of between 20 and 60 words and were developed in collaboration with a special educations teacher. Text predictability has not been assessed in this study. See table 4 for further details about the texts.

**Figure:**
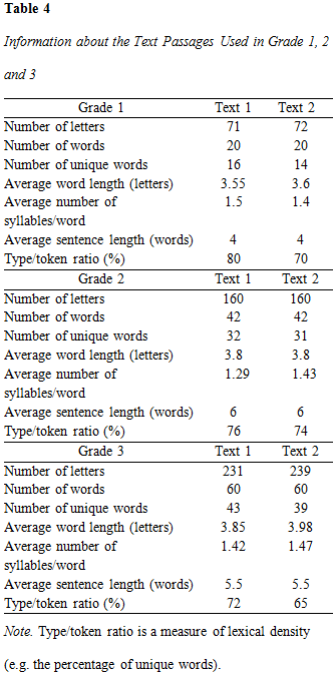


### Analysis

The eye movement analysis was performed using data from the text reading task. Prior to analysis the raw data from the eye movement recordings was exported to the software Optosphere (Version 2.1). The software provides an output consisting of eye movements classified as events, depending on their properties. A fixation is defined as the event of the eye remaining within an area corresponding to the fovea for at least 50 ms. Saccades are classified according to whether they occur within or outside the perceptual span and are identified as progressive (45-224 degrees) or regressive (224-45 degrees) depending on their directional angle. In the statistical investigation, saccades were analysed in terms of directional angle, amplitude and proportion of regressive saccades (27).

The data was filtered according to two criteria. First, extreme values were identified using Tukey’s interquartile range approach. Outliers were defined as values outside of the 1.5*IQR and were excluded from further analysis. Second, subjects were excluded if the difference in reading speed (WPM) between the two text conditions exceeded the outlier threshold values (n = 136). Analysis indicated technical issues accounted for discrepancies above this limit. After filtration, 2679 participants remained in total. Letter and Word Chains were excluded shortly after starting data collection in 2016, due to time restrictions. In total, 1448 participants completed Letter Chains and 1445 completed Word Chains.

Shapiro-Wilk’s W test was used to test the assumption of normality. The result rejects the null hypothesis of a normal distribution within all eye movement parameters and reading assessment measures (p < .05). However, W was close to 1 in all cases, indicating that deviations from normality were very limited. The sample size bias associated with Shapiro-Wilk test means that large sample sizes tend to result in a rejection of the null hypothesis, even though the deviation from normality may be trivial (41; 11). Given the sample size and high W values in the current analysis, further parametric testing was deemed applicable.

Letter and Word Chains were scored in accordance with the instruction manual. The scores constitute all correctly marked strings of letters and words. RAN was measured in letters per minute (LPM) and total naming time in seconds. Word and pseudo word reading were calculated by subtracting the number of misread words from the total amount of read words. Self-corrections were not marked as errors. The number of read words per minute (WPM) for text A and B was combined and averaged in the analysis of reading speed. A MANOVA was applied to examine the variance in the distribution and the effect of grade on eye movement and reading assessment variables. Pearson’s R was used to investigate all correlations. All statistical calculations were performed in R Studio (version 1.0.143). Mixed effects models were built to address the multilevel structure of the data. Models were set up separately for each test of the word reading assessment and the eye movement variables were inserted as fixed variables.

## Results

The descriptive data and analyses of variance for reading eye movements and reading assessment test results are reported separately, followed by the results of mixed effects modelling.

### Eye movements during text reading

Descriptive data for the eye movement parameters fixation duration (ms), forward saccade amplitude (°) and proportion of regressions is presented in table 5.

**Figure:**
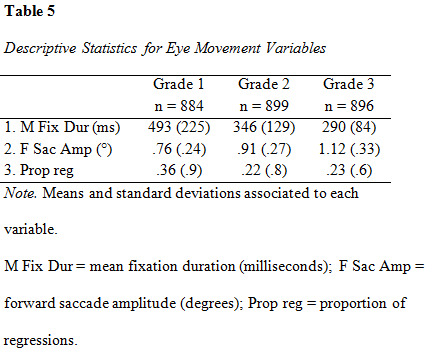


Values for each grade are listed separately. A MANOVA revealed a significant effect of grade on all three eye movement measures, Pillai’s Trace = .50, F(1,2663) = 870, p < .001. The average increase in fixation duration between the groups was 102 milliseconds, however the difference was almost 100 milliseconds larger between grade 1 and 2 (147 ms), as compared to between 2 and 3 (56 ms). Forward saccade amplitude increased with 0.18 degrees between the groups. The difference between grade 2 and 3 was slightly larger (0.21°) than between grade 1 and 2 (0.15°). Proportion of regressions decreased between grade 1 and 2 (14 %) while the difference between grade 2 and 3 was less noticeable (1 %). However, the direction of change between grade 2 and 3 was unexpected as regressions usually decrease with age. The average age of students in third grade is 9.75, which is slightly below the age of when values associated with skilled, adult reading typically are reached (31, 32; 3; 4).

All intercorrelations were significant (p < .001) except the associations between fixation duration and proportion of regressions and forward saccade amplitude and proportion of regressions, respectively, in grade 2 (see table 6). The strongest associations were yielded between mean fixation duration and forward saccade amplitude in second and third grade (r = .46 and r = .45, respectively).

**Figure:**
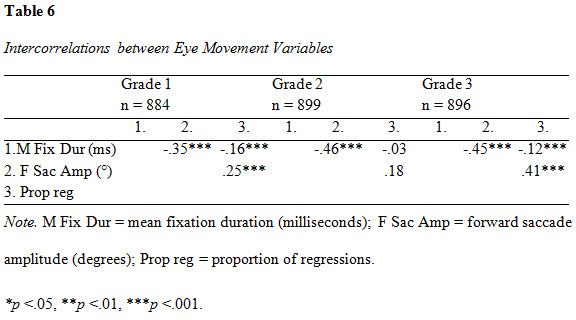


For the sake of graphical comparison and visualization of the distribution within the sample, eye movement parameters are plotted in figure 1, 2 and 3.

**Figure:**
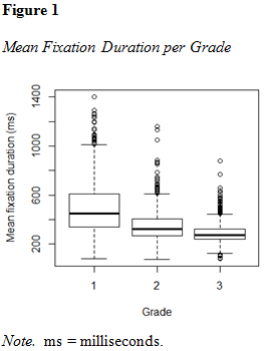


**Figure:**
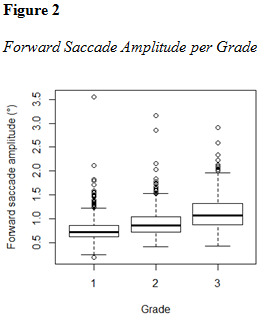


**Figure:**
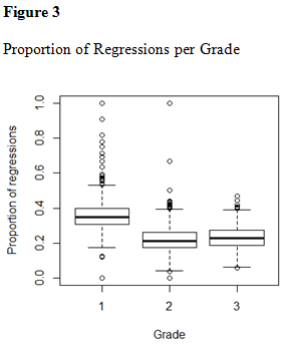


### Reading assessment

Descriptive data and intercorrelations from the analysis of reading assessment measures are presented in table 7, 8, 9 and 10.

**Figure:**
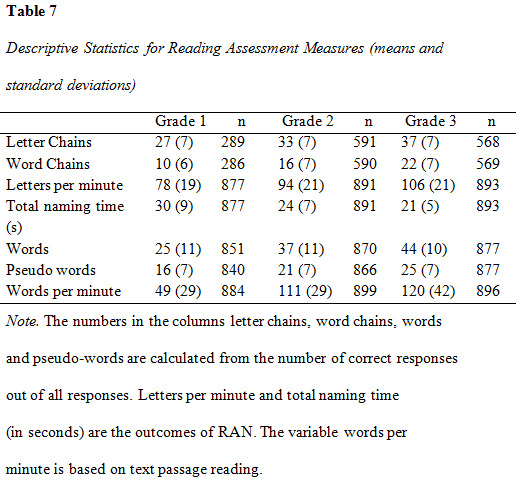


**Figure:**
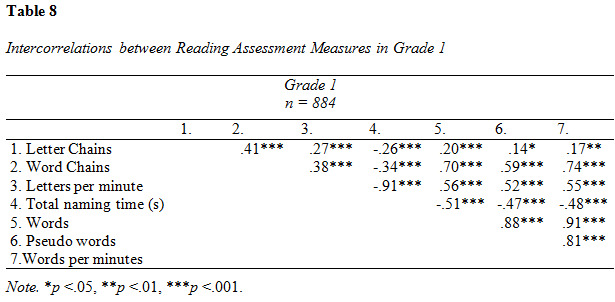


**Figure:**
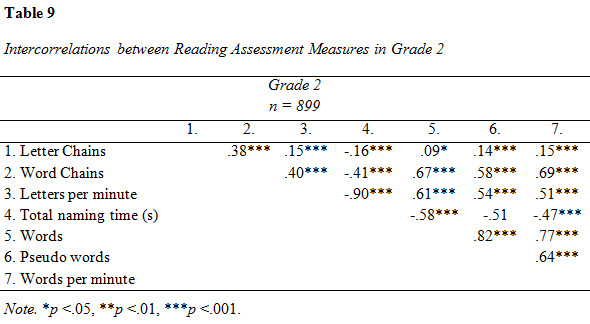


**Figure:**
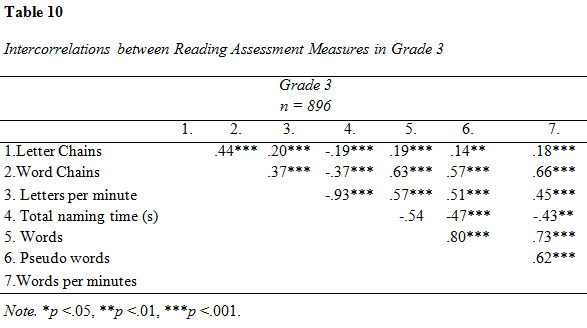


**Figure:**
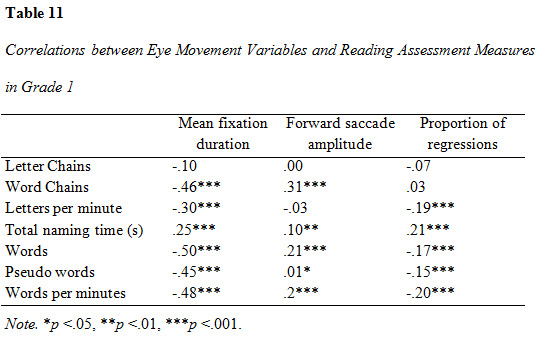


**Figure:**
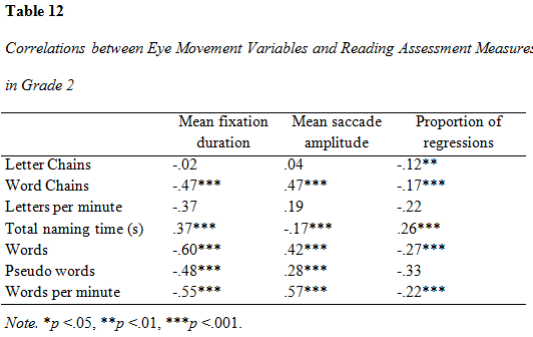


**Figure:**
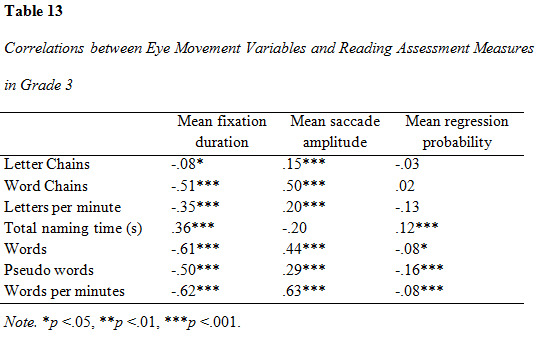


A MANOVA revealed significant effect of grade on all reading assessment scores, Pillai’s Trace = .40, F(1, 1388) = 128.4 p < .001, showing that results improved significantly with rising grade. The development of reading skill was particularly evident regarding reading speed during text passage reading; the participants in second grade read more than double the amount of words per minute than the first graders. The intercorrelations between reading assessment measures were overall moderate to strong (see table 8). Letter Chains was weakly correlated to the all other tests except Word Chains, to which the association was moderate.

### Correlation analysis of eye movement parameters and reading assessment measures

Correlations between eye movement measures and reading assessment results are presented in table 9, 10 and 11. All measures were entered in the correlation analysis. The analysis yielded predominantly significant results (α = .05). Several correlations were significant on the .01 and .001-level. The correlation between mean fixation duration and all word-based measures (word decoding, word reading and reading speed) was moderate to strong in all three groups and increased with rising grade. The strongest association was found between mean fixation duration and reading speed in grade 3. The strength of association between forward saccade amplitude and all reading assessment variables increased across groups, with the greatest difference between grade 1 and 2. Proportion of regressions had mostly weak correlations to the reading assessment measures. There was an observable increase in the strength of correlations between proportion of regressions and reading assessment outcomes in grade 2, although they were overall weak.

### Mixed effects analysis

Given the structure of the data (students nested in grades) we considered it suitable to build the model by inserting the fixed factors mean fixation duration, forward saccade amplitude and proportion of regressions as well as a random factor on the level of the grade (first, second or third) that the participant attended (see table 14). The marginal r^2^ was calculated based on the method described by Nakagawa and Shielzeth (2013), where the variance of the fixed effects is divided by the total variability (the sum of the variance of the fixed effects, the variance of the random effect and the variance of model residuals). The model estimates for the fixed variables were larger than their associated errors in all models, meaning their effect is distinguishable from zero.

**Figure:**
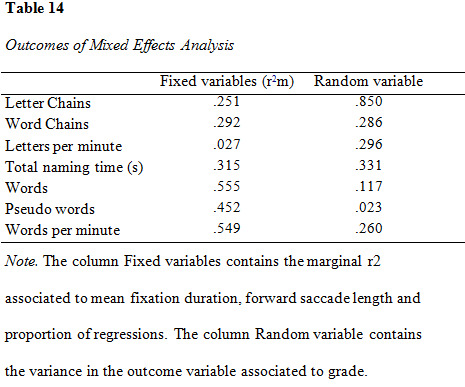


The models reach statistical significance in regard to each test investigated. The analyses yielded similar results, in terms of the direction of the effect associated to the fixed variables, irrespective of the outcome variable. In each model, an increase in fixation duration and regression probability was associated to a decrease in the outcome variable. In parallel, an increase in forward saccade length was associated to an increase in the outcome variable.

### Eye movements and Letter Chains

The fixed variables accounted for 2.7 % of the variance in Letter Chains test result when controlling for grade. Grade accounted for 30 % of variance. Increased mean fixation duration and proportion of regressions were associated to a lower test score, while increased saccade length also was associated to a higher result.

### Eye movements and Word Chains

The fixed variables accounted for 32 % of variance when controlling for grade, which in itself accounts for 33 % of variance. Increased mean fixation duration and proportion of regressions were associated to a lower test score, while increased saccade length was associated to a higher result.

### Eye movements and letters per minute

The fixed variables accounted for 25 % of the variance in the RAN measure letters per minute, when controlling for grade. Approximately 8.5 % of the variation was associated to the random effect. Increased mean fixation duration and proportion of regressions were associated to fewer letters named per minute, while increased saccade length was associated to a higher test result.

### Eye movements and total naming time

The fixed variables accounted for 29 % of the variance in the RAN measure total naming time, when controlling for grade. Approximately 2.9 % of the variation was associated to the random effect. Increased mean fixation duration and proportion of regressions were associated to longer naming time, while increased saccade length was associated to shorter naming time.

### Eye movements and word reading

The fixed variables accounted for 56 % of the variance in word reading. Approximately 12 % of the variation was associated to grade. Increased mean fixation duration and proportion of regressions were associated to a lower test score, while increased saccade length was associated to a higher result.

### Eye movements and pseudo word reading

The fixed variables accounted for 45 % of the variance in pseudo word reading. Approximately 2.5 % of the variation was associated to grade. Increased mean fixation duration and proportion of regressions were associated to a lower test score, while increased saccade length was associated to a higher result.

### Eye movements and text reading

The fixed variables accounted for 55 % of the variance in text reading (words per minute). Approximately 26 % of the variation was associated to grade. Increased mean fixation duration and proportion of regressions were associated to fewer words read per minute, while increased saccade length was associated to a higher result.

## Discussion

The purpose of this eye tracking study was to investigate the association between three basic eye movement variables and the outcomes of a multipart reading assessment on the basis of the data we have collected. We recorded eye movements during normal text reading in sample of Swedish school children in first to third grade. Mean fixation duration, forward saccade amplitude and proportion of regressions were extracted for analysis. The reading assessment included alphabetical rapid naming, decoding of letters and words, word and pseudo word reading, and reading speed.

In summary, results showed a significant decrease in mean fixation duration in parallel with a significant increase in mean forward saccade amplitude with higher grade. Proportion of regressions decreased between first and second grade, and increased slightly between second and third grade (1 %). Performance on all reading assessment tests improved significantly with rising grade. Fixation duration was robustly correlated to word-based reading assessment outcomes (Word Chains, word reading, reading speed) in all grades. The strength of association between forward saccade amplitude and word-based measures of reading ability grew across the grades. The results of linear mixed effects models showed that eye movements account for variability in reading assessment outcomes to a varying degree depending on the test in question. Grade accounted for a greater part of variance in test outcomes when the test content is different per grade, with the exception of alphabetical RAN which might be due to ceiling effects.

### Eye movements during reading

By and large, the developmental trends in reading eye movements showed similar patterns as previous research (31; 5; 4). However, mean fixation duration is longer in all grades than in several earlier studies, as is the range (ms) between grades (31). This tendency may be related to the naturalistic experimental set-up, affecting the precision of eye movement measures, and to sample characteristics. Children in first grade with a very basic reading skill as well as children with Swedish as a second language participated in this study, which might account for the distribution. Previous studies have found that long, ambiguous and infrequent words yield longer fixation times (18; 33), reflecting the processing difficulty experienced by the reader (3). Although the linguistic aspects of the text passages are not further analysed, beginning readers likely experienced greater difficulty processing the text than the readers with longer exposure to reading instruction and/or to Swedish. Moreover, previous studies vary with regards to stimuli and experimental design, which complicate comparisons. Variation in stimuli, for instance using word lists vs. paragraphs or tasks with unlimited time vs. time-limited, likely has effects on eye movement outcomes.

In light of the findings reported by Spichtig et al. (43), the comparison of our results and those reported in several previous studies is interesting. Notably, when comparing two large samples of children recorded in 1960 and 2011, Spichtig et al. found that present-day elementary school students assume what appears to be a sub-lexical processing strategy for a longer time than the students who participated in 1960. The present day students had significantly lower reading rates over grades, made more frequent fixations and decoded less text per fixation than their 1960 counterpart. Their findings suggest a decline in word recognition automaticity, evident both in the reported reading rates and eye movements. A comparative, longitudinal investigation of children’s reading and reading eye movements could uncover whether a similar development is actually present in young Swedish readers.

Nevertheless, the developmental changes demonstrated in the present study share many similarities with those reported in previous experiments, irrespective of task type and difficulty (18, 5; 6; 40). Saccade length typically increases gradually with age (32; 47), as was the case in this study. This has been accounted for by the development of the perceptual span, interrelated to improved lexical skill and shorter fixation duration (31). The decrease in proportion of regressions between first and second grade is another reflection of improved reading accuracy, as it means the second graders re-read less than the first graders (4). The decrease subsides between grade 2 and 3 which could be explained by at least two factors. The stability of proportion of regressions between second and third grade may reflect the rapprochement of adult levels in reading eye movement behaviour, which is normally reached at approximately 11 years of age (4). Alternatively, the text passages may have caused greater challenge to the participants in third grade. Further analysis of the texts’ linguistic attributes will be of interest to shed light to this matter. It is of importance to take into account that each grade was assigned different texts, which compromises the possibility of comparisons between groups. On the other hand, letting all participants read the same texts, irrespective of grade, would cause disproportionate difficulty for the youngest readers or ceiling effects among the older readers, depending on the text difficulty. Further data analysis using word-based eye movement parameters could indicate the level of reader processing difficulty associated to the unique texts.

### Reading assessment

The results from the reading assessment reflect improved reading skill, with significantly higher scores on all measurements, with rising grade. The increase in reading speed between first and second grade is likely the result of a generally improved word reading, which other test results corroborate. It points to an important developmental period regarding word reading ability between the first and second year of formal education. Letter Chains was only weakly correlated to the other assessment test scores, apart from Word Chains. Letter Chains largely assesses other abilities than the remaining tests, but poses similar demands on visual and motor skills as Word Chains which might account for their associations. However, Word Chains require word-level decoding skills while Letter Chains does not require the child to decode but rather to recognize similar characters. The remaining tests only requested verbal answers, and are thus less dependent on psychomotor skills. However, Word Chains results generally had strong correlations to the word based measures (word reading and reading speed), and slightly weaker to pseudo word reading. Rapid naming was a robust correlate to other measures of reading skill, especially to word reading, pseudo word reading and reading speed. Moreover, analysing the performance in terms of letters per minute had stronger correlations to these, than total naming time, which could be of clinical interest. Although, it should be noted that total naming time was a stable correlate to other test outcomes as well, with only slightly lower r-square values than letters per minute.

### Correlation between eye movement variables and reading assessment

The word-based reading assessment measures stand out regarding their relationship to eye movements, especially to mean fixation duration. Shorter fixations during text reading was associated with increased reading speed (in the same text reading task) and with higher results on other word reading tasks, including pseudo word reading. These correlations increased in strength across the grades. Together with the decrease in mean fixation time across grades, they may reflect the lessened cognitive load that is associated with improved reading skill (32; 19). Letter Chains and RAN overall had weaker correlations to eye movement parameters, possibly because of their format. Previous studies have found that word reading and RAN is a reliable predictor of eye movements during reading (23) and further inferential analysis could provide more information on the connection between rapid naming and eye movement outcomes in our sample.

A common decoding strategy among beginning readers involves decoding each letter sequentially (phonological decoding), with the goal of later automatization and orthographic sight reading. Consequently, the beginning reader’s forward saccades are shorter than the skilled readers’ and may leap between virtually each letter (4), a pattern that is similar to that of the saccades in individuals with RD (9). Both beginning readers and individuals with RD tend to allocate their cognitive resources to the fixated word and their perceptual span is consequently smaller (19). This induces shorter forward saccades and the first graders in this sample likely decoded letter by letter to a higher extent than second graders, whether they had RD or not. Efficient decoding and lexical processing of text are of growing importance with age, as the level of difficulty increases.

### Mixed effects analysis

The fixed variables accounted for a relatively large degree of the variance in most of the reading assessment tests, in particular in those assessing word-level decoding skill (word-chains, pseudo-words, real-words, and words per minute during text reading). The largest association was found for the real-word reading assessment, where the eye movement measures explained 56% of the variance in the outcome scores. Eye movements only explained a small part of the variance in letter chain results. Letter chains is a visual perceptive fine motor skill test rather than decoding test, and eye movements reflecting the reading process should therefore only be weakly associated to it.

In tests which contained differing test protocols depending on grade a larger part of variance was accounted for by grade, which had been entered as a random variable in our models. This pattern can be considered self-explanatory, as the discrepancy in test contents should produce some detectable variation in the results.

### Conclusions

In contrast to most previous studies on children’s reading, we attempted to scale up the number of participants included to get a large, diverse, and ecologically valid sample of eye movement data. However, this attempt in scaling has a number of implications that need to be taken into account when considering the results. First, this study examined eye movements in a situation that was, as far as possible, adapted to imitate a normal reading situation rather than an experimental situation. For example, eye movements were recorded in the children’s normal daily environment, i.e., in school rather than in an eye tracking lab. To enable a high-throughput of students, simplicity was a high priority. Thus, for example, eye movements were recorded without interfering with the natural behaviour of the participants (e.g. children were free to move their heads). Consequently, ecological validity is increased at the cost of precision and a greater margin of error than what is commonly seen in eye tracking research on reading.

In the present study, we have described the association between eye movements during reading and word reading ability, over a time period which is of great importance for children’s reading development. Our results provide additional evidence that there is a robust association between word reading ability and eye movements in children (4; 3; 35). Likely, the changes in eye movement measures reflect the underlying development of skills that are vital for successful reading. To understand their individual importance, future research might further investigate the contributions of these skills to variations in eye movement measures and over-all reading ability. Moreover, longitudinal studies are of interest to make predictions about reading skill based on earlier eye movement recordings in children. Finally, word-based eye movement measures are vital to the analysis for a more in-depth understanding of the development of children’s eye movement characteristics during reading.

## Ethics and Conflict of Interest

Authors GÖS and MNB own equity in a company whose aim is to offer new technologies for the assessment of reading deficits in school-age children. The venture is part of a project funded by Sweden’s innovation agency and the competing interest was disclosed to and approved by the Central Ethical Review Board (Ö 13/2015).

The author(s) declare(s) that the contents of the article are in agreement with the ethics described in http://biblio.unibe.ch/portale/elibrary/BOP/jemr/ethics.html and that there is no conflict of interest regarding the publication of this paper.

## Acknowledgements

This work was supported by Sweden’s innovation agency, VINNOVA, under Grant 2014-03459; the Sigvard and Marianne Bernadotte Research Foundation for Children’s Eye Care and Ulla and Ingemar Dahlberg’s Foundation.

The authors express their gratitude to the Sigvard and Marianne Bernadotte Research Foundation for Children’s Eye Care, Ulla and Ingemar Dahlberg’s Foundation, VINNOVA – Sweden's Innovation Agency and Trosa and Järfälla schools and municipalities for their support and contribution to this project.

## Appendix


