# Supplementary material for: Eye Movements during Reading and their Relationship to Reading Assessment Outcomes in Swedish Elementary School Children
Source: J Eye Mov Res. 2022 Oct 13;15(4):10.16910/jemr.15.4.3. doi: 10.16910/jemr.15.4.3 (PMC10205180; doi:10.16910/jemr.15.4.3)
Supplement: Supplementary file 1 [file jemr-15-04-c-SD1-01.pdf]

Appendix A

Word reading assessment

This appendix consists of the test sheets for Rapid Automatized Naming, word reading, pseudo word reading and text reading. Please, note that Letter Chains and Word Chains are not included here due to copyright matters.

Figure A1

Rapid Automatized Naming

s	a	k	c	n	t	a	t	n
k	c	s	n	t	s	a	c	k
n	a	k	c	t	s	t	a	k
n	c	s	a	n	c	k	s	t

Note. All participants were presented with the same set of letters, irrespective of grade.

Figure A2

Word Reading

en	på	är	av	om	vi	så	nu
att	det	som	för	med	den	har	kan
inte	vara	hade	alla	över	bara	även	utan
säger	under	efter	eller	andra	sedan	många	något
skulle	kommer	mycket	kronor	första	mellan	kanske	gäller
senaste	behöver	innebär	började	däremot	ungefär	månader	betyder
tidigare	dessutom	tillbaka	berättar	personer	nämligen	familjen	alldeles
människor	samtidigt	verkligen	framtiden	möjlighet	uppgifter	fortsätta	anledning

Note. All participants were presented with the same set of words, irrespective of grade.

Figure A3

Pseudo Word Reading

im	pu	ir	ov	un	vö	su	lu
ott	dyt	sön	fär	nyd	dym	hir	kum
ylti	vårå	hodi	ommo	ävir	boro	ivyl	atol
segir	aldir	iftir	immir	oldro	sidol	nulgo	lugåt
skammi	kånnir	nöckit	krålår	färsto	nimmol	kolski	gemmir
silosti	bihävir	ylliber	bärjodi	derinåt	algifer	nulodir	bitödir
tydygori	dissatån	tymmboko	birettor	pirsålir	lenmygil	fonymjil	ommdimis
nellyskår	sontydygt	virkmygil	frontydil	näjmyghit	appgyftir	fårtsetto	omlidlyng

Note. All participants were presented with the same set of pseudo words, irrespective of grade.

Figure A4

Text Passage for Grade 1

Lisa vill ha en kaka.

Kakor är goda.

Mamma har bakat.

Lisa ber om en kaka.

Men mamma säger nej.

Figure A5

Text Passage for Grade 1

Nina ska på kalas.

Det är på lördag.

På kalas är det roligt.

Man får tårta.

Man leker och busar.

Figure A6

Text Passage for Grade 2

Stina har en katt som heter Hugo.

Hugo tycker om att bli klappad.

Då njuter han. Hugo tycker också om

mat. Det bästa han vet är fisk. När

Hugo får fisk blir han mycket glad.

Hugo vill helst ha fisk varje dag.

Figure A7

Text passage for Grade 2

Jag har en kanin som heter Sotis.

Sotis är svart och har långa öron.

Sotis bor i en bur i mitt rum. En dag

rymde Sotis. Då blev jag ledsen.

Vi hittade Sotis hos min granne.

Sotis gömde sig under deras trappa.

Figure A8

Text Passage for Grade 3

Sara ska gå på bio idag. Hon ska se en ny film. Filmen är tecknad. Den handlar om en flicka som blir superhjälte. Sara ska se filmen med sin kompis Tobias. De handlar godis. Det ska de äta när de ser på filmen. Filmen är rolig. Flickan i filmen är modig. Hon åker ut i rymden. Hon räddar hela världen.

Figure A9

Text Passage for Grade 3

Ville går i skolan. Han lär sig många saker. Han lär sig läsa. Han lär sig räkna. Idag snöar det. Snön är vit. Då kan man åka pulka och kasta snöboll. Man får inte kasta snöboll på rasten. Gör man det blir lärarna arga. Ville vill kasta snöboll. Ville önskar att det vore lov för då kan inte lärarna bestämma.
